# Effect of hydrogen passivation on the decoupling of graphene on SiC(0001) substrate: First-principles calculations

**DOI:** 10.1038/s41598-017-09161-w

**Published:** 2017-08-16

**Authors:** Kang Liu, Pinglan Yan, Jin Li, Chaoyu He, Tao Ouyang, Chunxiao Zhang, Chao Tang, Jianxin Zhong

**Affiliations:** 1 Hunan Key Laboratory of Micro-Nano Energy Materials and Devices, Xiangtan University, Hunan, 411105 P. R. China; 2 Laboratory for Quantum Engineering and Micro-Nano Energy Technology and School of Physics and Optoelectronics, Xiangtan University, Hunan, 411105 P. R. China

## Abstract

Intercalation of hydrogen is important for understanding the decoupling of graphene from SiC(0001) substrate. Employing first-principles calculations, we have systematically studied the decoupling of graphene from SiC surface by H atoms intercalation from graphene boundary. It is found the passivation of H atoms on both graphene edge and SiC substrate is the key factor of the decoupling process. Passivation of graphene edge can weaken the interaction between graphene boundary and the substrate, which reduced the energy barrier significantly for H diffusion into the graphene-SiC interface. As more and more H atoms diffuse into the interface and saturate the Si dangling bonds around the boundary, graphene will detach from substrate. Furthermore, the energy barriers in these processes are relatively low, indicating that these processes can occur under the experimental temperature.

## Introduction

Graphene has attracted intensive research interest due to the potential applications in nanoelectronics, gas detection, and energy storage^1^. The exploitation of graphene-based devices crucially depends on the large-scale graphene production. Recently, homogeneous, large area and high quality monolayer graphene was successfully synthesized by epitaxial growth on the wide band gap semiconductor SiC(0001) surface^2–5^. This way has been widely regarded as the most promising ways for producing graphene wafers for electronic applications because SiC electronic devices are able to work at high voltages, high temperatures and high switching frequencies^6^ and are more convenient to integrate into existing device technology^7, 8^.

However, the first carbon layer is connected to the SiC substrate by covalent bonds with the $(6\sqrt{3}\times 6\sqrt{3})R{30}^{\circ }$ reconstruction, which is often called a buffer layer (BL)^9, 10^. The covalently bonded between SiC and graphene seriously affects the properties of graphene. No linear dispersion of π bands typical for free standing graphene is observed for this buffer layer^4^. The influence of the covalent bonding in the interface layer is also one of the primary suspects for the strongly reduced mobility in epitaxial graphene on SiC(0001) as compared to exfoliated graphene flakes, probably due to the introduction of scattering centers into the graphene layer^2–4, 11, 12^. Therefore, it is of crucial importance to remove the interface bonding and create quasi-freestanding layers for the practical application of epitaxial graphene on SiC(0001).

Many attempts have been made aiming at the detachment of the BL from the SiC substrate. Except for rapid-cooling technique^13^, atom intercalation is widely studied to detach the BL from the SiC. Riedl *et al*.^14^ firstly demonstrated that the bonds between the BL and the SiC substrate can be eliminated by hydrogen intercalation, and the BL is detached from the SiC substrate and displays the linear π bands as quasi-free-standing graphene monolayer. Similar results were obtained by Virojanadara *et al*. they exposed graphene-SiC samples to atomic hydrogen fluxes at temperatures higher than 450 °C^15^. Other experimental groups have also reported decoupling BL by intercalation of other elements such as fluorine^16^, oxygen^17–19^, germanium^20^, gold^21^ and so on.

To understand the mechanisms of atoms intercalation into graphene-SiC interface, many theoretical studies have been performed. Based on first-principle calculations, it is found that the energy barrier for H_2_ and H diffusion through the BL are very high (~6.5 eV^22^ and 4 eV^23^, respectively), and there are also no experimental evidences for hydrogen penetration through graphene^24, 25^. These results indicate that hydrogen is difficult to directly pass through the graphene to decoupling the BL. Therefore, it is possible that hydrogen intercalation occurs at grain boundaries or defects on the surface^26, 27^. A. Markevich *et al*. have modelled diffusion of hydrogen through defective graphene layers using the NEB method^27^. Their results showed that hydrogen atoms (molecules) diffusion through the hollow defects in graphene layer by overcoming a low energy barrier. It is further found that hydrogen can easily migrate along the graphene-SiC interface, break covalent bonds between the BL and the substrate, and passivate the SiC surface. However, H intercalation from the boundary into the graphene-SiC interface is still poorly understood. In this work, we have systemically investigated the process of H intercalation through the graphene zigzag edge and found that the passivation of H on both graphene edge and SiC surface plays crucial role in the decoupling process.

## Results and Discussion

**Figure 1:**
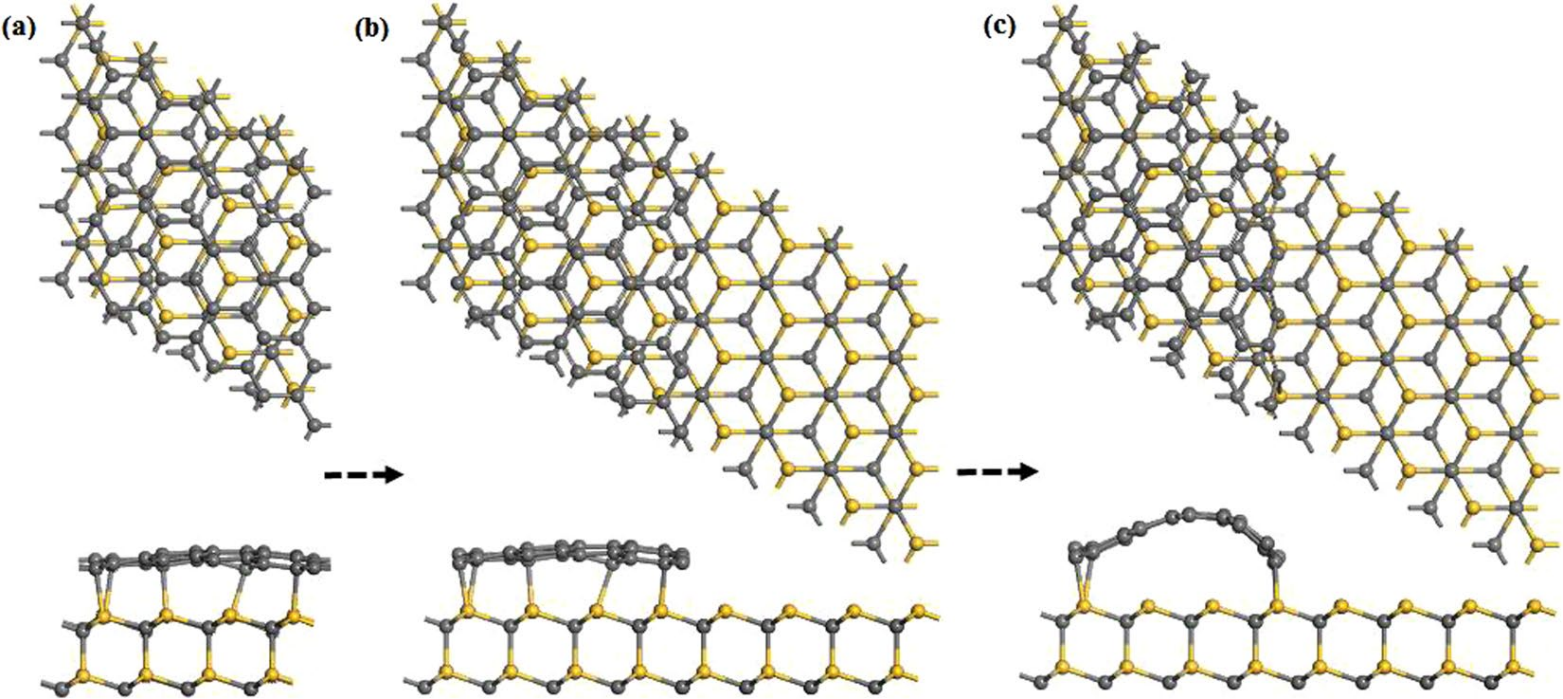
(**a**) Atomic structure of a (5 × 5) graphene placed on the (4 × 4) SiC (0001) surface. (**b**) and (**c**) Are the structures of graphene with zigzag edge on SiC substrate before and after relaxation, respectively.

To explore the BL decoupling from SiC, a (5 × 5) graphene was placed on the (4 × 4) 6H-SiC (0001) surface with about 1% mismatch. The vacuum separation (>2.0 nm) was used to be to avoid spurious interactions due to the periodic boundary condition. After relaxation, graphene is rippled and covalently bound to the substrate, as shown in Fig. 1(a). In order to investigate H intercalation from graphene edge, we then cut graphene into zigzag ribbon because zigzag edges are more stable than the armchair edges^28, 29^, as shown in Fig. 1(b). In our simulations, H atoms intercalated from the right side of graphene edge and the left edge is fixed on the substrate. After optimizing the structure, the right edge of graphene is bent downward and bonded to the SiC surface, as show in Fig. 1(c). In order to estimate the stability of geometry, we have investigated different geometries by shifting the ribbon with respect to the substrate and found that the structure in Fig. 1(c) is energetically favorable. Furthermore, the curved graphene and graphene nanoribbons on SiC substrate have been reported in previous experimental^30–33^ and theoretical^34, 35^ studies.

**Figure 2:**
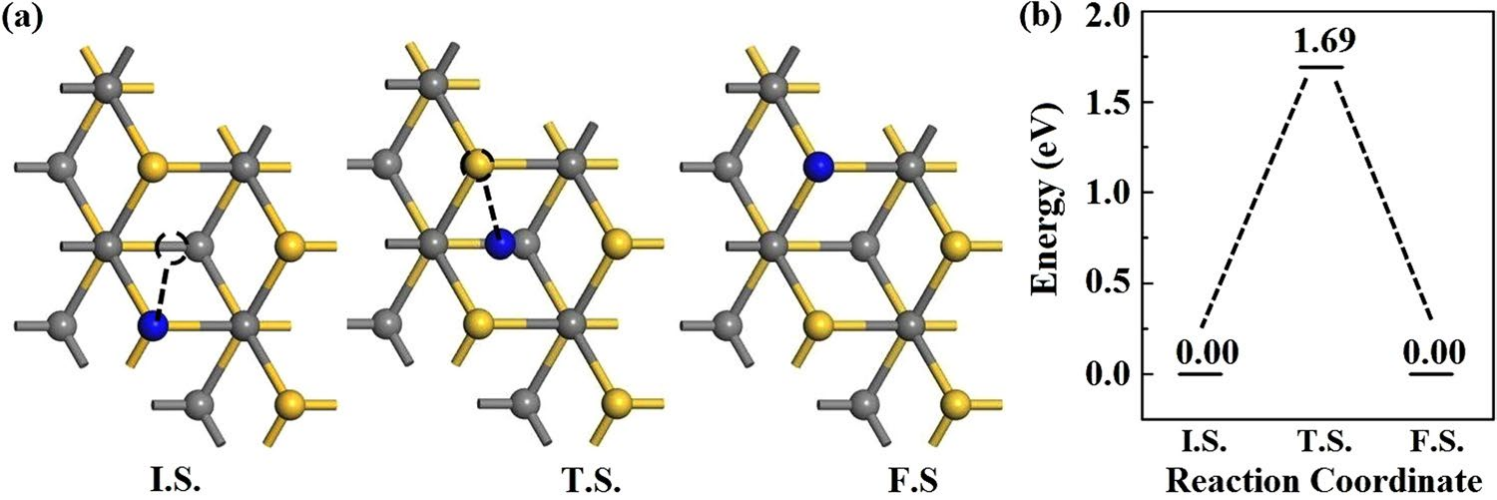
Migration pathway (**a**) and energy barrier (**b**) for the diffusion of H atom on SiC (0001) surface. The blue balls denote the H atoms, the gray balls denote the C atoms.

Firstly, H_2_ dissociation and H diffusion on SiC have been studied. It is found the hydrogen molecule spontaneously decomposes into two H atoms after adsorbing on the SiC surface. The migration path and energy barrier is shown in Fig. 2. One can see that H diffuses on SiC surface by moving through the hexagonal center. The energy barrier of H diffusion on SiC substrate is 1.69 eV, which means that H atoms are difficult to diffuse on the SiC surface at room temperature. Based on transition-state theory, ${{\rm{10}}}^{{\rm{12}}}\exp (-{E}_{{\rm{bar}}}/{k}_{{\rm{B}}}T)\approx {\rm{1}}$, where *E*_bar_ is the reaction barrier and *k*_*B*_ is the Boltzmann constant^26^ and the pre-exponential factor 10^12^ *S* ^−1^ can be viewed as the lower limit of the *KT*/*h*, the estimated temperature is about 700 K, which is in reasonable agreement with the experimental value^14, 15^. Thus the hydrogen atoms can be spread on the SiC surface at the certain temperature.

**Figure 3:**
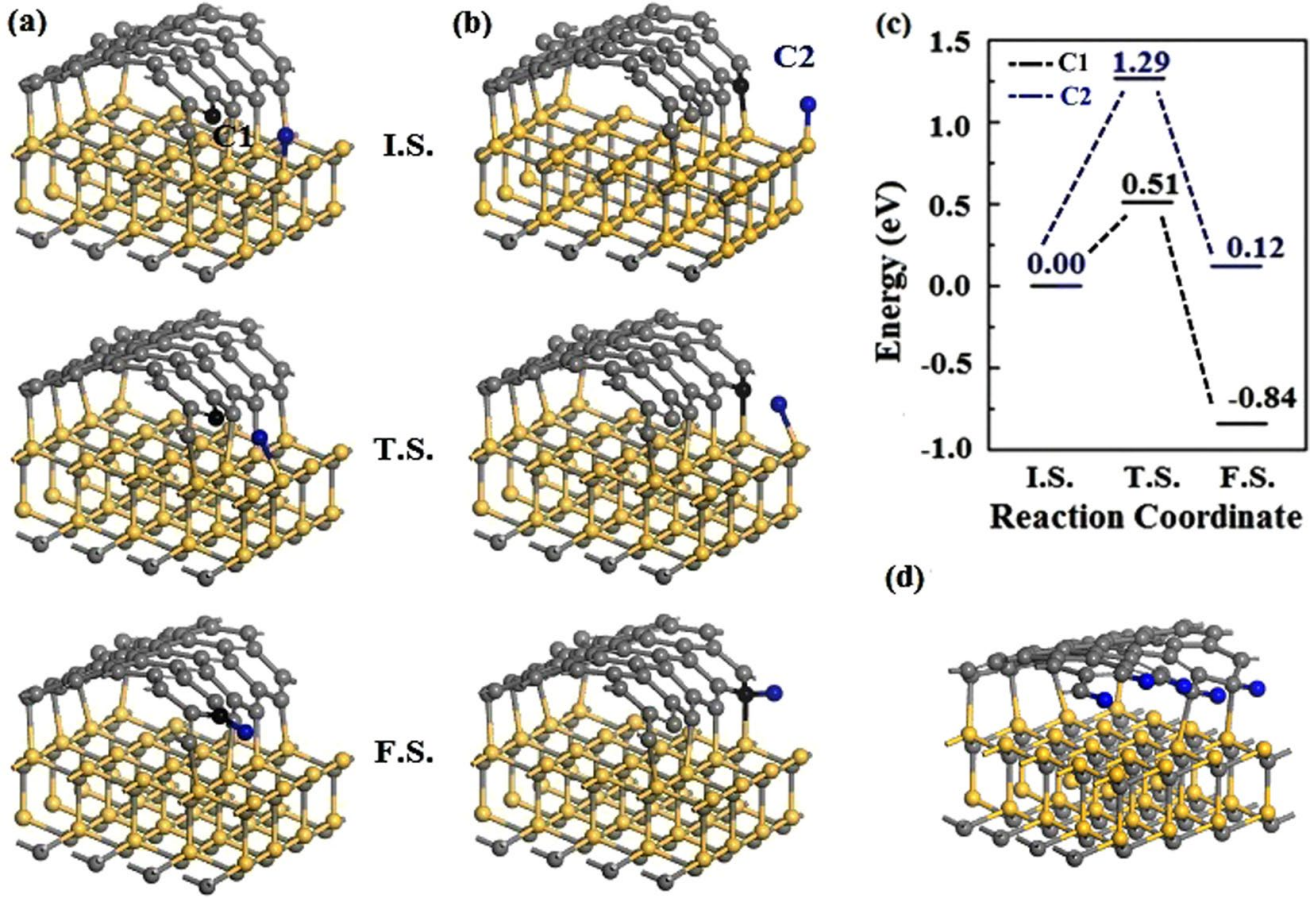
Reaction pathways for H diffusion to the site C1 (**a**) and C2 (**b**) on graphene edge. (**c**) Energy barriers for H diffusions. (**d**) Atomic structure of SiC and graphene system with full passivated graphene edge. The blue balls denote the H atoms, the black and gray balls denote the C atoms.

As shown in Fig. 1(c), carbon atoms on the ribbon edge are not all bonded with SiC substrate, thus the graphene edge is active and easy to be passivated by H atoms. Therefore, we then studied the process of graphene edge hydrogenation. There are two type of C atoms on the zigzag graphene edge, as denoted by C1 and C2 in Fig. 3. C1 atom is not bonded with Si atom, while C2 atom is bonded with Si atom on the substrate with a 1.845 Å Si-C bond. Figure 3(a) and (b) show the H diffuses from SiC substrate to C1 and C2, respectively, and the energy barriers are shown in Fig. 3(c). It can be seen that the energy barriers are low. Especially for H passivation on the C1, the energy barrier is 0.51 eV with a relatively large energy drop of 0.84 eV between the final state and the initial state, indicating that the process is energetically favorable. The energy barrier of H passivation on C2 is higher than that of C1, due to the two-coordinated C1 is more active than the three-coordinated C2.

In addition, the absorption energy are calculated to investigate the stability of hydrogen passivation on SiC and graphene edge. For H passivation on graphene edge, as shown in Fig. 3(d), the average absorption energy is obtained by $\overline{{E}_{{\rm{ads}}}^{\mathrm{SiC}/({\rm{G}}+{\rm{H}})}}=[{E}_{{\rm{tot}}}^{\mathrm{SiC}/\mathrm{HG}}-{E}_{{\rm{tot}}}^{\mathrm{SiC}/{\rm{G}}}-{E}_{{\rm{tot}}}^{{\rm{H}}}]/n$, where ${E}_{{\rm{tot}}}^{\mathrm{SiC}/G}$ is the total energy of the SiC and graphene ribbon system, ${E}_{{\rm{tot}}}^{\mathrm{SiC}/\mathrm{HG}}$ is the total energy of the system with the right edge of graphene fully passivated by H, ${E}_{{\rm{tot}}}^{{\rm{H}}}$ is the energy of H atom and *n* is the number of H passivation on graphene ribbon edge. For one H atom absorption on the SiC substrate, the absorption energy is obtained by ${E}_{{\rm{ads}}}^{(\mathrm{SiC}+{\rm{H}})/{\rm{G}}}={E}_{{\rm{tot}}}^{(\mathrm{SiC}+{\rm{H}})/{\rm{G}}}-{E}_{{\rm{tot}}}^{\mathrm{SiC}/{\rm{G}}}-{E}_{{\rm{tot}}}^{{\rm{H}}}$, where ${E}_{{\rm{tot}}}^{(\mathrm{SiC}+{\rm{H}})/{\rm{G}}}$ is the total energy of an H atom absorption on SiC substrate. The difference of the adsorption energies (${\rm{\Delta }}{E}_{{\rm{ads}}}=\overline{{E}_{{\rm{ads}}}^{\mathrm{SiC}/({\rm{G}}+{\rm{H}})}}-{E}_{{\rm{ads}}}^{(\mathrm{SiC}+{\rm{H}})/{\rm{G}}}$) is −0.41 eV, indicating that the system with graphene edge passviated is more stable. According to the energy barriers and the H gas environment in experiments, graphene edge can be passivated by H easily.

**Figure 4:**
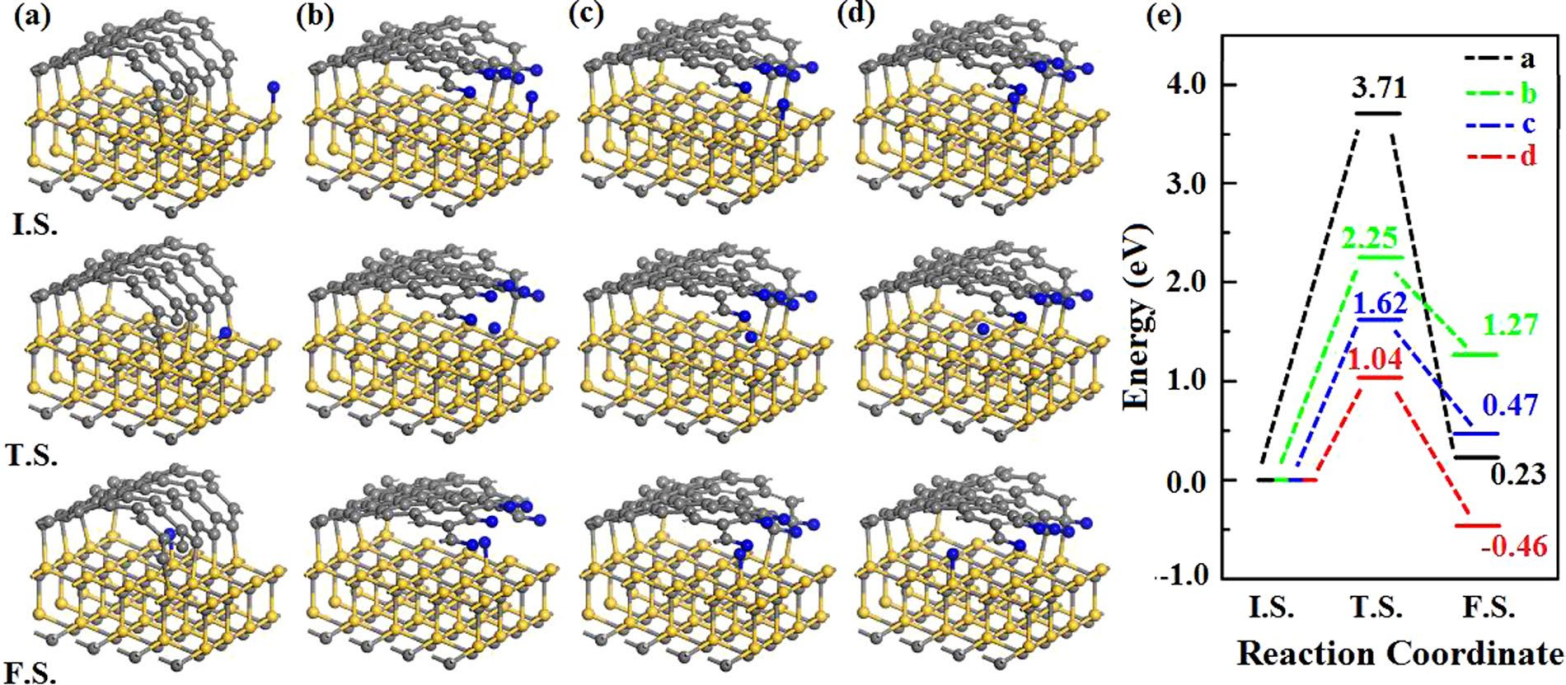
Reaction pathways for H atom intercalation into interface from (**a**) bare graphene edge, (**b**) from passivated graphene edge by breaking covalent bond and (**c**) from passivated graphene edge without breaking covalent bond. (**d**) Reaction pathway for H atom diffusion under the interface. (**e**) Energy barriers of H diffusion in (**a**,**b**,**c**) and (**d**).

Diffusion of H atoms into the SiC-graphene interface is the critical step for the decoupling process. To understand this process, we have considered three different diffusion paths for H migration into SiC-graphene interface. Firstly, the graphene edge is bare and H atom directly penetrates into the interface from the right side of Si-C bonds, and the reaction barrier is very high (3.71 eV) with an energy raise of 0.23 eV, as show in the Fig. 4(a) and (e). This means that it seems to be unrealistic for H atoms to directly cross the bare boundary, due to the graphene edge is strongly bonded with the Si atoms on the substrate. Secondly, the graphene edge is saturated by H and H atom breaks the Si-C bond at the boundary, as shown in Fig. 4(b). The energy barrier of this process is 2.25 eV and the total energy of the final state is 1.27 eV higher than the initial state, as show in the Fig. 4(e), indicating that breaking bond is also very difficult. Thirdly, the graphene edge is saturated and H atom crosses the interface without breaking Si-C bond, it is found that the reaction barrier is 1.62 eV, which is much lower than that of the other two cases. These results show that passivation of graphene edge can reduce the energy barrier significantly and H tends to diffuse into the interface without breaking Si-C bonds. Furthermore, we have investigated the H atom diffuse along the SiC surface below the interface graphene layer, it is found that the reaction barrier is 1.04 eV with an energy drop of 0.46 eV, as show in the Fig. 4(d) and (e). Thus hydrogen atoms can intercalate between H-passivated graphene and SiC surface, and migrate along the SiC surface to saturate the dangling bonds of SiC substrate.

**Figure 5:**
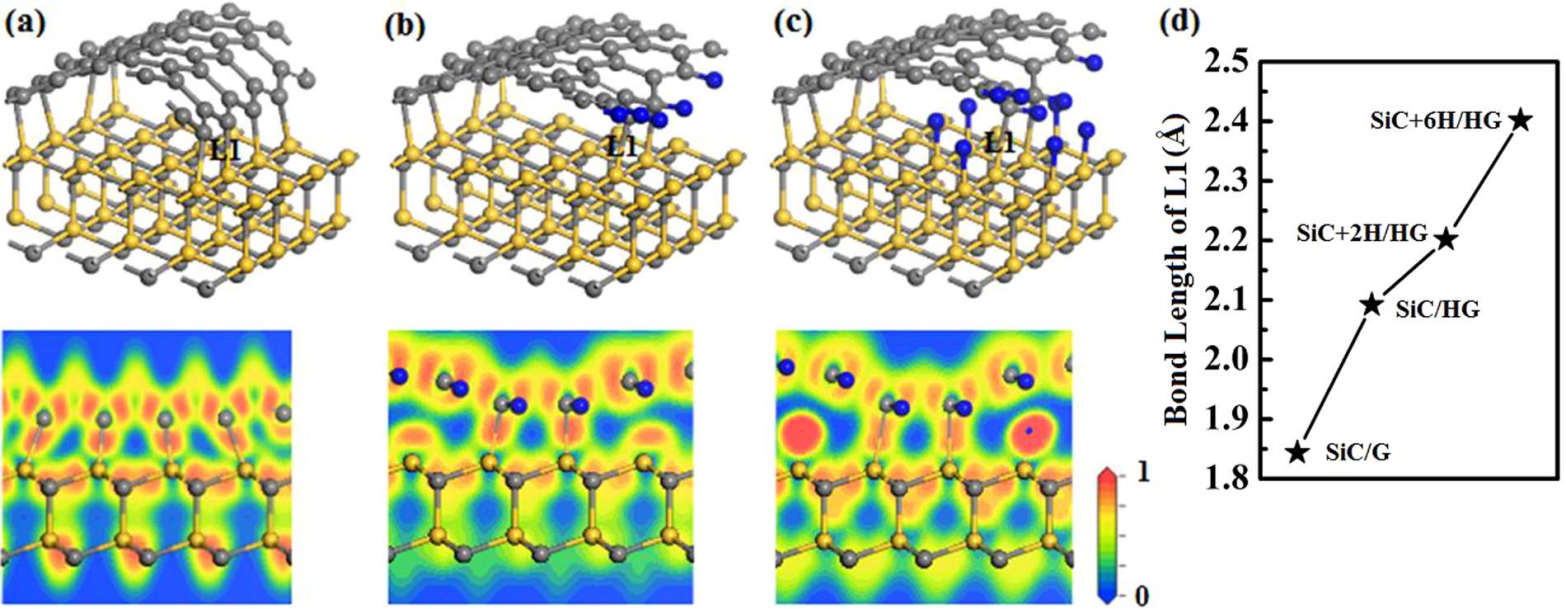
Atomic structures (upper panels) and ELFs (lower panels) of SiC/G (**a**), SiC/HG (**b**) and SiC + 6 H/HG (**c**), respectively. (**d**) Variation of bond length L1 versus H passivation. (SiC + 2 H/HG means two H atoms adsorb on the interface boundary).

After H passivation on graphene edge, graphene is still bonded with SiC substrate by two Si-C bonds. To further understand the decoupling of graphene and SiC substrate, we investigate the decoupling by increasing the number of H diffusion into the graphene-SiC interface, as shown in Fig. 5(c). The electron localization function (ELF) and the length of Si-C bond (L1) are calculated to illustrate the variation of the interaction between graphene and SiC substrate during H passivation. For comparison, the ELFs of the systems without H passivation on graphene and SiC (SiC/G), with H passivation on graphene (SiC/HG) and with H passivation both on graphene and SiC (SiC + 6 H/HG) are presented in Fig. 5(a,b and c), respectively. As shown in Fig. 5(a), there are four Si-C bonds and a local maximum (attractor) locates between Si and C atoms of each bond, indicating the strong covalent bonding. For SiC/HG, one can see that there are only two Si-C bonds and the region of the high electron localization is elongated, reflecting weakening of the covalent bonding due to H passivation on graphene edge. For the system SiC + 6 H/HG, there are six H absorbed on SiC substrate and the local maximum of ELF is almost split into two local maxima, indicating that the covalent binding is nearly broken. Figure 5(d) shows the variation of the length of Si-C bond. For SiC/G, the bond length is 1.844 Å, which is similar with the bond length of bulk SiC. After H passivated graphene edge (SiC/HG), the bond length is 2.092 Å. When SiC substrate is saturated by two and six H atoms, the bond length are 2.202 Å and 2.402 Å. The change of bond length also indicates that the interaction between graphene edge and SiC substrate is weaker as H passivation on SiC and graphene edge, which is in agreement with ELF.

**Figure 6:**
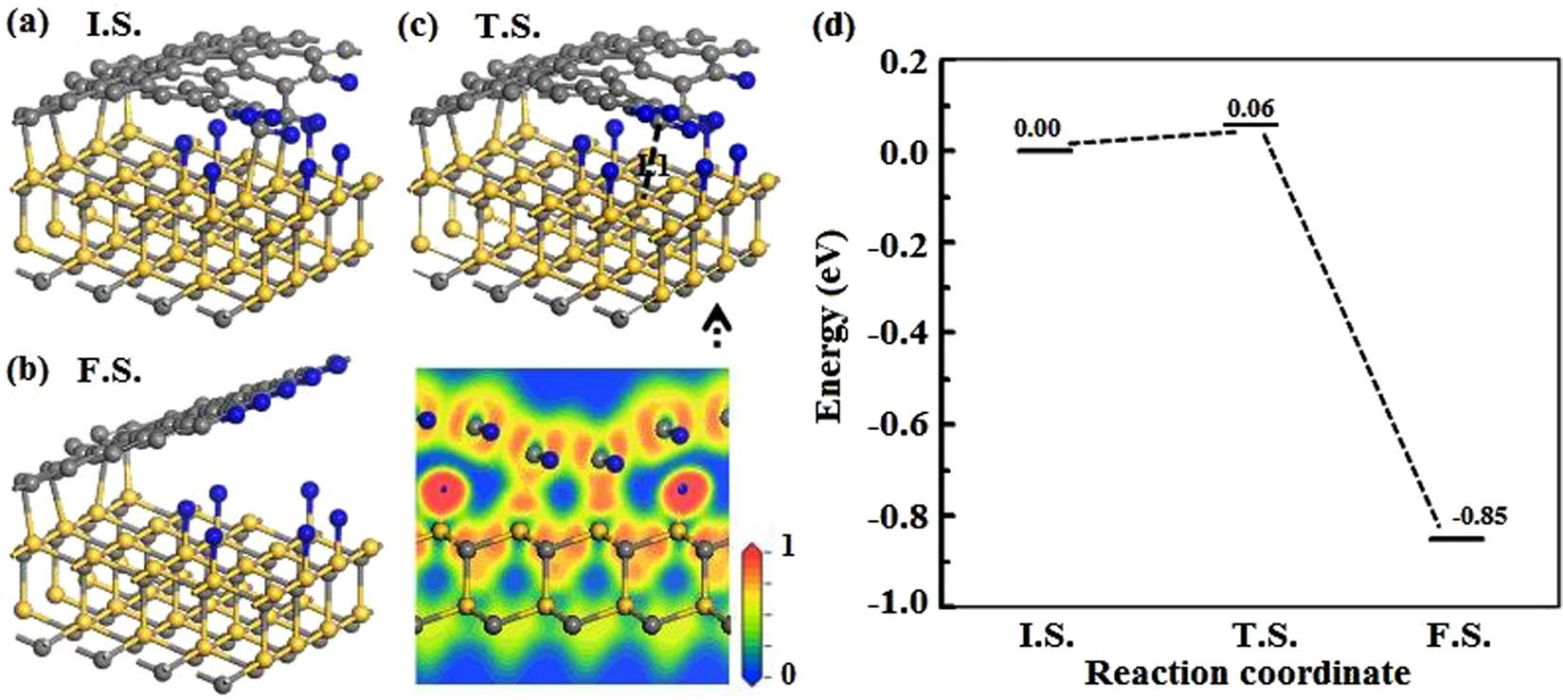
Migration pathway (**a**,**b**,**c**) and energy barrier (**d**) for the graphene edge decoupling from the SiC (0001) surface. (**a**) and (**b**) Are the initial state (I.S.) and final state (F.S.). (**c**) Is the atomic structure and ELF of the transition state (T.S.).

Based on the system (SiC + 6 H/HG), we have calculated the reaction path and the energy barrier for graphene completely decoupling from SiC substrate, as shown in Fig. 6. In the final state, the right side of graphene is totally decoupled with SiC surface, which is similar to the results of previous study^36^. It is found that the energy barrier is very small (~0.06 eV) and the total energy of final state is 0.85 eV lower than the initial state, thus this process would happen very easily in experiments. From the ELF of the transition state in Fig. 6(c), one can see that there are two local maxima between Si and C atoms, which means the covalent bond is almost broken. These results suggest that graphene can decouple from SiC substrate from the boundary, which is consistent with the experiments^16, 37^.

### Summaries

In summary, we have systematically studied the decoupling of graphene from SiC surface by H atoms intercalation from graphene boundary. Our results suggest that the decoupling process can be divided into the following steps. Firstly, H_2_ molecular decompose into two H atoms on the SiC surface and H atoms diffuse on the substrate. As C atoms on the edge are active, H atoms will saturate on graphene edge, which will weaken the interaction between graphene and the substrate. Finally, graphene will detach from SiC substrate as more and more H atoms saturate the Si dangling bond around the boundary. It is found that the H passivation on both graphene edge and SiC substrate is the key factor to decoupling graphene from the substrate. Furthermore, the energy barriers in these processes are relatively low, indicating that these processes can occur under the experimental temperature.

## Methods

Density functional theory (DFT) calculations were performed using the Vienna Ab Initio Simulation Package (VASP)^38^ with the projector-augmented wave^39^ (PAW) approach to represent the electron–ion interaction. The exchange and correlation functional was treated by the generalized gradient approximation of Perdew–Burke–Ernzerhof (PBE)^40^. The energy cutoff of the plane-wave expansion of the basis functions was set to be 400 eV and only Gamma point was used for the Brillouin zone integration. All atomic positions were fully relaxed using the conjugate gradient algorithm until all interatomic forces were smaller than 0.1 eV/nm. The climbing image nudged elastic band (CI-NEB)^41, 42^ method was employed to determine the transition states and activation barriers.
